# Retrotransposons and Diabetes Mellitus

**DOI:** 10.3390/epigenomes8030035

**Published:** 2024-09-06

**Authors:** Andromachi Katsanou, Charilaos Kostoulas, Evangelos Liberopoulos, Agathocles Tsatsoulis, Ioannis Georgiou, Stelios Tigas

**Affiliations:** 1 Department of Endocrinology, University of Ioannina, 45110 Ioannina, Greece; a.katsanou@gni-hatzikosta.gr (A.K.); atsatsou@uoi.gr (A.T.); 2 Department of Internal Medicine, Hatzikosta General Hospital, 45445 Ioannina, Greece; 3 Laboratory of Medical Genetics, Faculty of Medicine, School of Health Sciences, University of Ioannina, 45110 Ioannina, Greece; chkost@uoi.gr (C.K.); igeorgio@uoi.gr (I.G.); 4 First Department of Propaedeutic Internal Medicine, Medical School, National and Kapodistrian University of Athens, Laiko General Hospital, 11527 Athens, Greece; elibero@med.uoa.gr

**Keywords:** DNA methylation, retrotransposons, LINE-1, Alu, diabetes mellitus, nuclear elements, global hypomethylation, epigenetic modifications

## Abstract

Retrotransposons are invasive genetic elements, which replicate by copying and pasting themselves throughout the genome in a process called retrotransposition. The most abundant retrotransposons by number in the human genome are Alu and LINE-1 elements, which comprise approximately 40% of the human genome. The ability of retrotransposons to expand and colonize eukaryotic genomes has rendered them evolutionarily successful and is responsible for creating genetic alterations leading to significant impacts on their hosts. Previous research suggested that hypomethylation of Alu and LINE-1 elements is associated with global hypomethylation and genomic instability in several types of cancer and diseases, such as neurodegenerative diseases, obesity, osteoporosis, and diabetes mellitus (DM). With the advancement of sequencing technologies and computational tools, the study of the retrotransposon’s association with physiology and diseases is becoming a hot topic among researchers. Quantifying Alu and LINE-1 methylation is thought to serve as a surrogate measurement of global DNA methylation level. Although Alu and LINE-1 hypomethylation appears to serve as a cellular senescence biomarker promoting genomic instability, there is sparse information available regarding their potential functional and biological significance in DM. This review article summarizes the current knowledge on the involvement of the main epigenetic alterations in the methylation status of Alu and LINE-1 retrotransposons and their potential role as epigenetic markers of global DNA methylation in the pathogenesis of DM.

## 1. Introduction

Epigenetics is defined as “mitotically heritable alterations in gene expression that do not directly change the DNA sequence” [1]. Epigenetic marks include (1) chromatin modifications (e.g., histone protein methylation, ubiquitination, acetylation), (2) noncoding small and long RNA (e.g., microRNA [miRNA], lncRNA and P-Element-induced wimpy testis (PIWI-interacting) RNA [piRNA]), and finally, (3) DNA modifications (e.g., DNA methylation or hydroxymethylation) [2]. The best characterized epigenetic modification is DNA methylation (DNAm) [3], “a covalent modification of DNA involving the transfer of a methyl group to the fifth carbon of a cytosine resulting in the formation of 5-methylcytosine by DNA methyltransferases” and occurs mainly in a CpG dinucleotide context [4]. DNAm is vital for regular development playing a significant role in various functions, such as genomic imprinting, regulation of tissue-specific gene expression, inactivation of the X-chromosome and suppression of repetitive element transcription and transposition [5]. Thus, irregular alterations in DNAm have been identified in several diseases [6].

Global DNAm can be estimated through repetitive genome elements, such as LINE-1 (long interspersed nucleotide element-1) and Alu element (the most abundant of short interspersed nucleotide elements, SINES), and is linked with genomic instability and chromosomal abnormalities in the regions of gene promoters. These regions can be either activated or silenced, depending on the methylation pattern [7]. Recent scientific evidence suggests that changes in the methylation status of retrotransposons (especially of LINE-1 and Alu) are the reasons for widespread hypomethylation and genomic instability in various types of cancer, autoimmune disorders, and diabetes mellitus (DM) [7,8,9,10]. Due to the growing application of whole-genome sequencing for diagnostic purposes and the fact that about one-third of genome methylation occurs in these elements, the significance of retrotransposons and their potential use as global markers in the pathogenesis and development of several conditions has recently emerged [11,12]. Even though previous research indicates that alterations in LINE-1 and Alu methylation are associated with DM [13], obesity-related disorders, and cardiovascular disease (CVD), the outcomes are still disputed, and the implicated processes have not been determined as yet [7].

Overall, the aim of this review article is to summarize the current knowledge on the involvement of the main epigenetic alterations in the methylation status of Alu and LINE-1 retrotransposons (which are the most abundant and active in the human genome) and their potential role as epigenetic markers of global DNAm in the pathogenesis of DM.

## 2. Retrotransposons (LINE-1 and Alu)

### 2.1. Structure

Retrotransposons are delineated in classes, such as short and long interspersed elements (SINEs and LINEs, respectively) and are divided into groups and subgroups based on sequence similarity and assumed copying origin [2,14]. Interestingly, they mobilize using a copy-and-paste mechanism through an RNA intermediate [15]. The most abundant retrotransposons by number in the human genome are Alu and LINE-1 elements, which comprise about 40% of the human genome [16,17]. There are approximately one million copies of Alu and more than 500,000 copies of LINE-1, which have the ability to replicate and integrate themselves into new positions throughout the human genome [10,18].

LINE-1 retrotransposons can move autonomously and encode the enzymatic machinery necessary for their transposition. LINE-1 exceeds 6000 nucleotides in length (nt) and exists in over 100,000 copies in the human genome, constituting 18% of the genome, although only approximately 100 LINE-1s are considered to be potentially active [10,19]. Specifically, the majority of LINE-1 element copies are inactive and frequently represented only by short 3′ end fragments [20].

**Figure 1:**
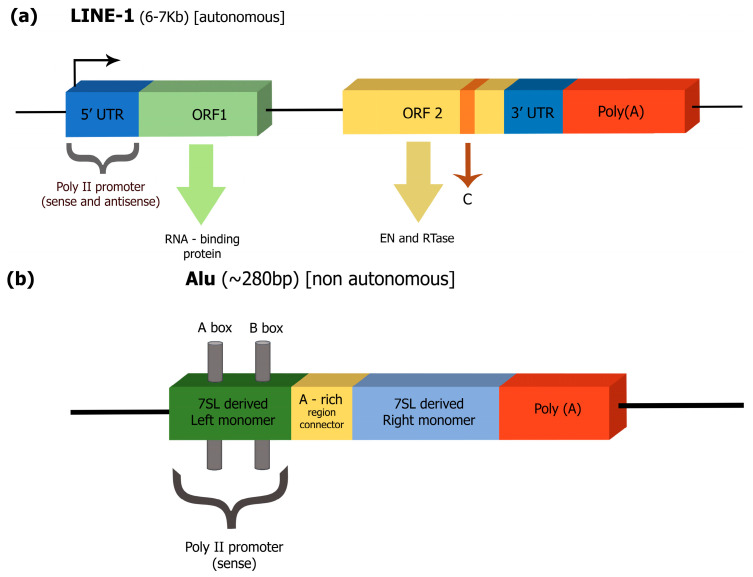
Examples of the structure of retrotransposons. (**a**) Structure of LINE-1: Autonomous elements (such as LINE-1) encode the protein activities essential for retrotransposition, such as a reverse transcriptase and an endonuclease. The arrow at the 5′ end of retrotransposons indicates the transcription start site from their internal promoter. (**b**) Structure of Alu: Nonautonomous elements (such as Alu) do not encode proteins and their retrotransposition relies on proteins encoded by autonomous elements [15]. ORF: open reading frame; UTR: untranslated-region sequences; EN: endonuclease; Pol II: RNA polymerase II promoter; Pol III: RNA polymerase III promoter (bars labeled A and B); C: denotes cysteine-rich domain (encoded by ORF2); A-rich: adenosine-rich (AR) linker.

Functional LINE-1 elements are almost 6–7 kb long, usually comprising two coding open reading frames (ORF1 and ORF2) and a long 5′ end with RNA polymerase II promoter activity [21]. ORF1 proteins play a role in identifying and transporting the RNA template into the nucleus, while ORF2 is responsible for encoding endonuclease (EN) and reverse transcriptase (RT) [10,22], [Figure 1a]. It is essential to mention that LINE-1s are also able to mobilize protein-coding RNAs [9].

On the other hand, non-autonomous elements, such as Alus, are usually trans-mobilized by the LINEs’ machinery [23,24]. The Alu element is a short ~300 nt retrotransposon, which does not produce its own transposition machinery. It consists of two divergent dimers, ancestrally derived from the 7SL RNA gene, which are separated by a short A-rich region. Alu’s 3′ end has an extended A-rich region which is crucial for its amplification process [25], [Figure 1b].

LINE-1 elements possess a high content of adenine and thymine (AT-rich), while Alu elements are rich in guanine and cytosine (GC-rich). This variation difference in base composition might have been selected to target distinct chromosomal regions [25]. The insertions are processed transcripts: the intron is removed and the 3′ end of the inserted LINE-1 is polyadenylated. On the other hand, the adenine tails of Alu insertions are shorter [26]. Active elements and the products of their insertion are commonly known as transposable and interspersed repetitive elements (TIREs) [27].

### 2.2. Function

The ability of retrotransposons not only to proliferate but also to inhabit eukaryotic genomes has made them evolutionarily successful. This is accountable for generating genetic modifications that result in substantial effects on their hosts [28]. The evolutionary timeline of these elements (the point at which they were inserted into the human genome) can be estimated via several sequence variants. For example, their incorporation in the eukaryotic genome can interfere with the expression of neighboring genes by causing disruptions in the regulatory sequences of exon–intron interactions [17,18].

Retrotransposons can be regulated both transcriptionally and post-transcriptionally [29]. During mammalian development, various epigenetic histone modifications happen to the DNA packaging protein histone H3. For example, H3K9me3 (indicating trimethylation of lysine 9 on histone H3 protein subunit), H3K9me2, and H3K27me3 supplement each other to suppress retrotransposons during global DNAm reprogramming [30]. Retrotransposons may also modify transcriptions via distinct mechanisms, serving as alternative promoters or enhancers. Transcripts of transposable elements (TE), which can be classified as either DNA transposons or retro (RNA)-transposons, can aid in the binding of Transcription Factors (TF) to the target genes. Additionally, the complementary enhancer RNA (eRNA), which is abundant with Alu sequences, can control the pairing of enhancer and promoter by forming a duplex with upstream antisense promoter RNA (uaRNA). Recent studies suggest diverse roles for eRNA, a category of non-coding RNAs (ncRNAs) transcribed from enhancer regions, revealing their potential clinical applications in human diseases, such as metabolic diseases, neurodegenerative disorders, cardiovascular diseases, and malignancies [31]. PIWI-interacting RNAs (piRNAs) are responsible for inhibiting the transposition of transposable elements in germ cells, thereby safeguarding the integrity of germline genomes, ref. [32] and PIWI–piRNA complexes identify transposon transcripts based on sequence matching, both in the nucleus and in the cytoplasm [30,33].

In the human population, only Alu, LINE-1, and SVA elements maintain the capability to transpose and so, they constitute the majority of TE insertion polymorphisms contributing not only to phenotypic variation, but also to disease susceptibility as well [34]. It is important to note that Alu elements are abundant in gene-dense regions, while LINE-1s are scarce in these areas. Furthermore, de novo insertions of these elements could disrupt proper gene function, resulting in highly penetrant phenotypes and monogenic diseases [9,35].

Both elements can contribute to disease development through various mechanisms. These include transpositional mutations by disrupting a gene, by causing homology-mediated deletions or by disruption of chromatin influencing the expression of adjacent genes [2]. The timing and reasons for the activation of retrotransposons remain a crucial question. Environmental stimuli, including specific infections, have been correlated with their triggering [10]. Epigenetic regulation can be influenced by the environment and DNA methylation in particular is especially sensitive to environmental perturbation during the early stages of gestation, when epigenetic patterns can be inherited across subsequent cell divisions. For example, differential DNA methylation is correlated with gestational exposures to toxicants, such as bisphenol A [BPA], arsenic or cigarette smoke [2].

### 2.3. Biological Role—Clinical Significance

Retrotransposons play a significant role in the chromatin structuring, and particularly, the transcriptions of LINE-1 and Alu are crucial for nuclear segregation [30,36]. It is worth mentioning that one-third of genome methylation occurs in these elements [7], so hypomethylation of retrotransposons is an essential factor promoting retrotransposition, especially during aging [2,17]. Consequently, quantifying LINE-1 and Alu methylation is considered to serve as a surrogate measurement of global DNAm level [13,37,38,39,40]. For example, it has been proposed that alterations in DNAm within LINE-1 or Alu elements could serve as potential novel biomarkers of early-stage dementia in patients with type 2 diabetes (T2D) [17]. Also, in a recent review of Evidence for Prenatal Epigenetic Programming on human populations, LINE-1 and Alu elements were used as epigenetic markers of global DNAm measures with an ELISA-based method [41].

Retrotransposons, as class I TEs, possess numerous traits that could render them useful as indicators or biomarkers of exposure and disease status: (1) they are extensively distributed across the entire genome, (2) they are not subject to selective constraint, and (3) they are commonly highly methylated, thus any alteration in their methylation status could indicate a significant environmental influence [2].

### 2.4. Methods for Methylation Analysis and Measure

Common methods for analyzing DNAm in humans involve quantifying DNAm levels at specific genes, retrotransposons, and across the majority or entirety of genes (epigenome-wide analyses) [2,42]. Numerous widely used techniques for assessing methylation of human transposable elements, such as LINE-1 and Alu, depend on sodium bisulfite treatment of the DNA (which converts unmethylated cytosine residues into uracil, while leaving methylated cytosines unchanged), followed by sequencing [43], methylation-specific quantitative polymerase chain reaction (PCR) [44], pyrosequencing [45], and finally, targeted bisulfite sequencing, which benefits from next-generation sequencing (NGS) technology [2,46]. Moreover, the combination of long-read and short-read sequencing methods in single-cell RNA sequencing (scRNA-seq) serves as an effective analytical tool for examining retrotransposon sequences in cell-specific transcriptomes [47]. In addition, by using RNA in situ conformation sequencing (RIC-seq), it has been revealed that retrotransposons, especially Alu elements, are abundant in interactions between enhancers, promoters, and RNA (known as EPRIs). These interactions play a crucial role in ensuring the proper pairing between enhancers and promoters [48].

The progress in genomic technologies, including scRNA-seq, RICseq, and Hi-C, has significantly enhanced our knowledge about the role of retrotransposons in the functions of host genomes and their evolutionary expansion [30]. Finally, dbRIP, a database of human Retrotransposon Insertion Polymorphisms (RIPs) contains all currently known polymorphic LINE-1 and Alu insertion loci [10,49,50].

### 2.5. Correlation with Diseases

DNAm serves two fundamental purposes: prevention of genomic stability and gene expression regulation [51]. Thus, dysregulation of epigenetic patterns can lead to diseases, such as cancer, diabetes, neurological disorders, infectious diseases, and autoimmune conditions [1]. Even though most LINE-1 and LTR promoters are suppressed in healthy cells, they can be reactivated in disease states, particularly in tumors and transformed cells [34].

Specifically, changes in the methylation status of Alu and LINE-1 have been observed in placental development and aging [52,53], exposure to certain environmental factors and nutritional deficiencies [2], neurodegenerative diseases, multiple sclerosis, autism spectrum disorders [54,55,56,57], autoimmune diseases [58,59], cancer [60], cardiovascular diseases [7,11], obesity, and diabetes [61,62,63,64].

## 3. Methylation of Retrotransposons (LINE-1 and Alu) and Diabetes Mellitus

According to current evidence, alterations in DNAm may contribute to the increasing incidence of DM [51,65,66,67]. During aging, there is an increase in global hypomethylation events, particularly in repetitive sequences, which are believed to trigger the reactivation of retrotransposon elements, like Alu and LINE-1. Consequently, these changes in the epigenome are observed in noncommunicable diseases associated with aging, such as DM [53,68]. For example, recent research demonstrated the connection between epigenetic processes and hyperglycemia or late complications in patients with type 1 diabetes (T1D) [69]. Specifically, prolonged high blood glucose levels led to aberrant epigenetic changes that persisted even after the normoglycemic environment was restored and maintained, suggesting the involvement of epigenetics in metabolic memory [5,70,71,72,73]. Moreover, recently, Štangar et al. summarized the major HLA loci associated with T1D (HLA region 6p21.32) for Alu and LINE-1 subfamilies (DQA1, DQB1, DRB1, Β), annotated by RepeatMasker (a program that scans DNA sequences for interspersed repeats) [10,74].

Previous studies revealed increased oxidative DNA damage and decreased DNA repair activity in DM, suggesting that individuals with T2D accumulate DNA damage [75]. DNA damage, genomic instability, and cellular senescence [76] leading to alterations in the methylation status of retrotransposons are related to the presence of prediabetes or T2D [51,77].

### 3.1. LINE-1 Methylation, Glucose Metabolism and DM

Many studies have related methylation of LINE-1 with an increased risk of abnormal carbohydrate metabolism, hyperglycemia, insulin resistance, obesity, and cardiovascular disease (CVD). However, results are controversial, as depicted below (Table 1), and limited to T2D, whereas according to our knowledge, scientific evidence about any possible correlation between LINE-1 methylation and T1D is missing.

Previous studies have indicated the potential involvement of epigenetic processes in T2D as a crucial interface between the impact of genetic predisposition and environmental effects. For example, a prospective cohort intervention study evaluated whether global DNAm, using LINE-1 methylation as indicator, could predict increased risk of carbohydrate metabolism disorders. In that study, LINE-1 methylation was quantified by pyrosequencing technology using two study groups: (a) those with a pre-existing disorder of carbohydrate metabolism (impaired fasting glucose, impaired glucose tolerance or T2D) at baseline, whose glycemic status improved one year later, and (b) individuals whose glycemic status remained the same or deteriorated after one year. Individuals with low baseline LINE-1 methylation levels exhibited a higher risk of T2D or impaired glucose metabolism (IFG, IGT, or both) during follow-up [77].

In the same research study, individuals whose glycemic status did not improve had lower levels of overall LINE-1 DNAm and LINE-1 hypomethylation correlated with an increased risk of metabolic status deterioration, regardless of other classic risk factors [78]. In a 14-year longitudinal cohort study of 794 patients with T2D, correlations between global LINE-1 DNAm status and specific metabolic markers prevalent in T2D [such as body mass index (BMI), HbA1c, blood pressure (BP), high sensitivity CRP, and lipid profile] were detected [79]. At baseline, a 10% rise in LINE-1 methylation was inversely associated with diastolic BP, eGFR, and cholesterol/HDL cholesterol ratio, but there was no association with HbA1c. In the same study, over the 14-year follow-up period, a 10% increase in LINE-1 methylation correlated with a reduction in BMI by 2.5 kg/m^2^ in women and in the cholesterol/HDL cholesterol ratio (by 0.7 mmol/L) [79].

Moreover, in a case-control study of 205 patients with T2D and 213 healthy controls, leukocyte telomere length (LTL) and the DNAm of LINE-1 were evaluated by quantitative PCR and quantitative methylation-specific PCR (qMSP), respectively [13]. The results highlighted a significant increase in LINE-1 methylation in patients with T2D compared to controls. Shorter LTL correlated with increased risk of T2D, while lower LINE-1 methylation levels were associated with a reduced risk of having the disease [13].

Pearce et al. observed an association between LINE-1 DNAm and risk factors for T2D and CHD, such as fasting glucose and serum lipid levels. LINE-1 positive associations were observed between log-transformed LINE-1 DNAm and fasting glucose, total cholesterol, triglycerides, and LDL–cholesterol concentrations, but a negative association was reported between log-transformed LINE-1 methylation, HDL cholesterol, and HDL/LDL ratio. The authors suggested that this correlation between global LINE-1 DNAm and both glycemic and lipid profiles highlighted a potential role for epigenetic biomarkers as predictors of metabolic disorders and T2D [80].

Furthermore, in a case-control analysis, reduced LINE-1 methylation in peripheral blood leukocytes was associated with the diagnosis of DM, aging, and increased risk of CHD. However, no statistically significant associations were noticed between LINE-1 methylation levels and BMI, homocysteine, triglyceride or diagnosis of hypertension [81]. Martín-Núñez GM et al. evaluated the effect of different bariatric surgery (BS) procedures (Roux-en-Y gastric bypass and laparoscopic sleeve gastrectomy) on global DNAm by studying the alteration of LINE-1 methylation status in two groups of severely obese patients with or without DM. No differences in LINE-1 methylation levels were observed at baseline and 6 months after BS in the two groups. However, at baseline, there was a positive correlation between LINE-1 methylation levels and weight in both obese patients with and without DM [82]. In another study, no notable differences in LINE-1 methylation between 14 obese individuals (OC) (with no established insulin resistance) and 24 patients with T2D were observed, although in the group with T2D, methylation had a tendency to increase over time [83]. Similarly, in a cross-sectional study involving 431 adolescents, no correlations were detected between LINE-1 methylation and obesity indicators (BMI, percent of body fat and waist circumference) in saliva cells [84]. However, in a cross-sectional study of 186 subjects (with or without metabolic syndrome), the estimated LINE-1 methylation levels in visceral adipose tissue cells were negatively associated with fasting glucose levels, the presence of metabolic syndrome (MS), and diastolic BP. Also, a strong correlation was found between LINE-1 hypomethylation and an elevated risk of MS in the presence of obesity [85].

**Table 1:** Effect of LINE-1 methylation in DM.

Reference/Year of Publication	Type/Duration of Study	Subjects	Biological Sample Used	Method/Area of Research	Main Findings
Martín-Núñez GM et al., 2014 [78]	Prospective cohort intervention study (exercise, and Mediterranean diet) 1 year	310 subjects: (a) 155 with a pre-existing carbohydrate metabolism disorder (IFG, IGT or T2D) and improved glycemic status after 1 year (b) 155 subjects whose glycemic status did not change or worsened after one year	WBC	LINE-1 DNAm was quantified by pyrosequencing	Subjects whose carbohydrate metabolism status did not improve showed lower levels of global LINE-1 DNA methylation. LINE-1 DNAm was linked with the risk of metabolic status worsening independent of other classic risk factors.
Malipatil et al., 2018 [79]	Longitudinal T2D cohort study 14 years (2002–2016)	794 T2D patients (60% M/40% W)	PB samples	Pyrosequencing (QIAGEN PyroMark Q96 MD pyrosequencer)/quantification of percentage LINE-1 DNAm	Increase in LINE-1 DNAm status was associated with reduction of specific metabolic markers in T2D (BP, BMI, eGFR, CHOL/HDL). No significant association with HbA1c.
Wu et al., 2017 [13]	Case-control study	205 T2D patients and 213 healthy controls	WBC	LINE-1 methylation was measured by quantitative methylation-specific PCR (qMSP)	LINE-1 was significantly hypermethylated in T2D patients.
Pearce et al., 2012 [80]	Case-control study	228 individuals (aged 49–51 years) with risk factors for T2D and CHD	PB samples	Pyrosequencing (PyroMark MD Pyrosequencer Qiagen, UK)/quantification of percentage LINE-1 DNAm	Positive associations between log-transformed LINE-1 DNAm and fasting glucose, TCHOL, TRG, and LDL. Negative association between log-transformed LINE-1 methylation and HDL and HDL:LDL ratio.
Wei et al., 2014 [81]	Case-control study	334 cases with CHD and 788 healthy controls	WBC	DNAm was estimated by LINE-1 repeats using bisulfite pyrosequencing	Reduced LINE-1 methylation was associated with diagnosis of diabetes, aging, and increased risk of CHD.
Martín-Núñez GM et al., 2017 [82]	Bariatric surgery intervention (BS)	60 patients (30 nondiabetic/30 with diabetes and severe obesity	WBC	LINE-1 DNAm was quantified by pyrosequencing	6 months after BS, no differences in LINE-1 methylation over time in the 2 groups (with or without diabetes). LINE-1 methylation was positively associated with body weight at baseline.
Remely et al., 2013 [83]	Controlled intervention: (GLP-1R agonists for T2D and nutritional counseling) 4 months	(1)14 obese individuals (OC) with no established insulin resistance, (2) 24 insulin-dependent T2D patients, and (3) 18 controls (normal weight)	WBC	Bisulfite conversion and Pyrosequencing	LINE-1 methylation was similar between the groups or the time points.
Turcot et al., 2012 [85]	Cross-sectional study	186 subjects (34 M and 152 F) Group 1: Without metabolic syndrome (MS) (14 M and 84 F) Group 2: With MS (20 M and 68 F)	Visceral adipose tissue cells	LINE-1 DNAm was quantified by pyrosequencing	LINE-1 methylation was negatively associated with fasting glucose levels, MS, and diastolic BP. LINE-1 hypomethylation was strongly associated with the increased risk of MS in the presence of obesity.
Carraro et al., 2016 [12]	Cross-sectional study	40 subjects (9 M and 31 F), BMI: 22.4 ± 3.4 kg/m^2^	PBMC	The quantitative analysis of LINE-1 and 3 gene promoters was determined after bisulfite treatment in a 7900HT Fast Real-Time PCR System	LINE-1 hypermethylation was positively associated with insulin resistance and markers of adiposity (BMI and WC).

Note. DNAm: DNA methylation; M: male; F: female; T2D: type 2 diabetes; BMI: body mass index; BP: blood pressure; MS: metabolic syndrome; IFG: impaired fasting glucose; IGT: impaired glucose tolerance; HbA1c: glucosylated hemoglobin A1c; BS: bariatric surgery; CHD: coronary heart disease; qMSP: quantitative methylation-specific; OC: obese individuals; MS: metabolic syndrome; PCR: polymerase chain reaction; WC: waist circumference; PB: peripheral blood; WBC: white blood cells; TCHOL: total cholesterol, TRG: triglycerides; HDL: high-density lipoprotein cholesterol; LDL: low-density lipoprotein cholesterol; PBMC: peripheral blood mononuclear cells.

Some researchers analyzed the relationship between methylation levels and insulin resistance with divergent findings. While Carraro et al. [12] noticed a positive association between the methylation of LINE-1 and HOMA-IR index, Piyathilake et al. [86] found an association between hypomethylation, increased HOMA-IR, and weight (especially in the presence of low folate concentrations). Specifically, in Carraro’s cross-sectional study, LINE-1 hypermethylation was found to be positively associated with insulin resistance, markers of adiposity (WC and BMI), and poor diet quality [12].

A previous report reviewing 14 observational (cross-sectional and longitudinal) and 6 interventional (diet, exercise and bariatric surgery) studies concluded that LINE-1 methylation correlated with body composition and obesity-related disorders, including T2D, insulin resistance, and CVD [7]. Evidence has so far been conflicting, as both negative and positive associations have been observed between LINE-1 methylation and cardiometabolic markers related to insulin resistance and T2D [87,88,89,90]. A number of observational studies evaluated the association between LINE-1 methylation and adiposity indices (body weight, body fat, BMI, and WC) or the risk of overweight-obesity. Three studies found a positive association [12,90,91], two studies found no significant association [84,92], and four studies found a negative association [85,86,93,94]. On the other hand, various intervention studies evaluated LINE-1 methylation before and after nutritional intervention, physical activity, and/or bariatric surgery. The relatively short follow-up period (ranging from 6–12 months) used in these studies to detect any changes in weight and metabolic benefits, was probably insufficient to observe alterations in DNAm or LINE-1 methylation, which could partly explain the negative findings [7,78,82,83,88,95,96].

Finally, a systematic review on the role of epigenetic modifications in cardiovascular disease evaluated 31 studies including 12.648 individuals and a total of 4037 CVD events [11]. Findings highlighted the epigenetic regulation of 34 metabolic genes associated with glucose and lipid metabolism, fetal development, inflammation and oxidative stress. Of those, three studies used LINE-1 methylation [81,97,98] and one study used Alu methylation [99] to examine global DNAm in blood samples. Global DNA methylation evaluated at LINE-1 was inversely associated with CVD, whereas a higher degree of global DNA methylation estimated at Alu repeats was associated with the presence of CVD [11].

### 3.2. ALU Methylation and DM

Despite numerous studies implicating Alu repeat elements in several diseases, there is sparse information available concerning the potential functional and biological role of these repeat elements in DM (Table 2). In particular, scientific data regarding their possible involvement in the development of T1D are limited.

For example, a previous study focused on the insertion/deletion polymorphism of the AluYb8-element in the *MUTYH* gene (*AluYb8MUTYH*), a genetic risk factor for T2D. Mitochondrial DNA (mtDNA) content and unbroken mtDNA were significantly increased in the mutant compared to the wild-type patients, although no association between mtDNA transcription and *AluYb8MUTYH* variant was noticed, suggesting that this variant was linked with an altered mtDNA maintained in patients with T2D [100].

A recent report indicated that enhancers in pancreatic beta cell loci are usually Alu retrotransposons, which are associated with T2D and are responsive to endoplasmic reticulum (ER) stress [101]. Based on this observation, Hansen et al. identified 91 genes through transcriptome-wide association studies (TWAS), whose expressions are associated with high waist-to-hip ratio adjusted for body mass index in women. Consequently, a massively parallel reporter assay (MPRA) was conducted revealing that most Alu sequences tested did not stimulate reporter gene expression in adipocytes. However, a subset of Alu elements demonstrated extremely high enhancer activity in pre-adipocytes, suggesting that some Alus may have been exapted as enhancers of nearby metabolism-related genes. The authors concluded that a subset of these enhancers might influence the distribution of body fat in women and increase insulin resistance by increasing the risk of T2D [102].

In another study, authors aimed to investigate the polymorphic nature of Alu DNA fragments in the human tissue plasminogen activator (tPA) gene in subjects with or without DM [93]. Genomic DNA was isolated from 76 patients with DM (26 with T1D and 50 with T2D) and 60 non-diabetic controls and the Alu fragment was amplified using PCR. The genotype of 80% of the non-diabetic subjects was (Alu−/−), whereas 36.8% of the diabetic patients exhibited the Alu−/− or Alu+/− genotype and 26.3% the Alu+/+ genotype. Thus, the Alu−/− genotype appeared less frequently in individuals with DM, suggesting that this deletion of the Alu fragment in the *tPA* gene might play a protective role against DM [103]. A recent case-control study focused on the frequency of Alu repetitive elements, insertion/deletion (I/D) polymorphism, in angiotensin-converting enzyme among diabetic retinopathy (DR) patients and whether this polymorphism is associated with the severity of retinopathy in patients with T2D. The presence of Alu repetitive elements did not elevate the development or progression risk of DR, whereas no association between I or D alleles and the severity of DR was detected [104].

**Table 2:** Effect of Alu methylation in DM.

Reference/Year of Publication	Type of Study	Subjects	Biological Sample Used	Method/Area of Research	Main Findings
Yasin et al., 2019 [103]	Case-control study	76 DM patients (26 with T1D and 50 with T2D) and 60 aged-matched healthy individuals	PB samples	DNA extraction. Alu fragment was amplified using PCR.	Significant protective effect of the Alu−/− genotype in the tPA gene against DM.
Walid et al., 2021 [104]	Case-control study	277 subjects (100 diabetic patients without DR, 82 diabetic patients with DR, and 95 healthy controls)	PB samples	DNA extraction. Alu repetitive elements were examined by PCR.	Alu element polymorphism did not affect the age of onset of diabetes in patients with or without DR.
Katsanou et al., 2023 [64]	Case-control study	36 patients with T1D and 29 healthy controls	PB samples	DNA extraction. DNAm levels and patterns of Alu were investigated by using the Alu-COBRA.	Total Alu methylation rate (mC) was similar between patients with T1D and controls. Patients with T1D had higher levels of the partial Alu methylation pattern (mCuC + uCmC). This pattern was positively associated with HbA1c and negatively with the age at diagnosis.
Thongsroy et al., 2023 [51]	Case-control study	203 subjects in 3 groups (56 normal controls, 64 pre-DM patients, and 83 T2D patients)	WBC	DNA extraction and Alu COBRA analysis.	Alu methylation in T2D patients progressively decreases with increasing HbA1c levels.
Thongsroy et al., 2017 [77]	Case-control study	240 subjects in 3 groups (80 normal controls, 80 with pre-DM, and 80 with T2D)	WBC	DNA extraction and Alu COBRA analysis.	In the DM group, Alu hypomethylation was directly associated with high FBS, HbA1C and BP.

Note: DNAm: DNA methylation; DR: diabetic retinopathy; T1D: type 1 diabetes; TD2: type 2 diabetes; Alu-COBRA: COmbined Bisulfite Restriction Analysis method; tPA gene: tissue plasminogen activator gene; BP: blood pressure; FBS; fasting blood sugar; ROS: reactive oxygen species; IL-1β: interleukin-1β; HbA1c: glucosylated hemoglobin A1c; PCR: polymerase chain reaction; PB: peripheral blood; WBC: white blood cells.

In another case-control study from our group, DNA methylation levels and patterns of Alu methylation were examined in the peripheral blood of 36 patients with T1D and 29 healthy controls by the ALU-COBRA method [95]. The total Alu methylation rate (mC) was similar between patients with T1D and controls, but patients with T1D were found to have significantly higher levels of the partial Alu methylation pattern (mCuC + uCmC). In addition, this pattern was found to be positively associated with the levels of HbA1c but negatively with the age at diagnosis. However, no correlation between the total methylation or Alu methylation patterns and the duration of disease or the presence of chronic diabetes complications was identified [64].

Furthermore, Thongsroy et al. measured the levels of Alu methylation in normal, pre-T2D, and T2D patients by the ALU-COBRA method. Alu methylation levels in the groups with DM were significantly lower compared to healthy subjects and this Alu hypomethylation was directly associated with fasting glucose, HbA1c, and BP [77]. Moreover, a few years later, Thongsroy et al. aimed to further investigate the longitudinal alterations in Alu methylation levels in patients with T2D. In addition to significantly decreased Alu methylation levels in patients with T2D compared to healthy controls, the investigators observed changes in Alu hypomethylation within the same individuals over a follow-up period. Finally, Alu methylation was inversely associated with elevated levels of HbA1c in patients with T2D [51].

Another research conducted on umbilical vein endothelial cells (HUVEC) showed that accumulation of endogenous Alu RNA during hyperglycemia provoked oxidative stress and dysfunction in these cells, by impeding the expression of superoxide dismutase 2 (SOD2) and endothelial nitric oxide synthase (eNOS) at transcription and translation levels via the NFκB signaling pathway. This information indicates that endogenous dsRNA homologous to the Alu Sc subfamily gathered in HUVEC cells under hyperglycemic conditions [105].

Finally, a genome-wide sequence analysis of 941 T1D candidate genes was performed to identify embedded Alu elements [39]. A notable enrichment of Alus within these genes was observed, highlighting their importance in T1D. Eight T1D genes have been identified harboring inverted Alus (IRAlus) within their 3′ untranslated regions (UTRs), which are known to control the expression of host mRNAs by generating double stranded RNA duplexes. Further analysis predicted the formation of duplex structures by IRAlus within the 3′UTRs of T1D genes, so it was suggested as the potential role of IRAlus in regulating the expression levels of the host T1D genes [39].

## 4. Discussion

In previous studies, as shown in Table 1 and Table 2, investigators usually preferred to assess global DNAm by using LINE-1 rather than Alu retrotransposons’ methylation as a marker. However, Alu retrotransposons are the most abundant and active in the human genome [2] and are primarily methylated in noncoding regions, providing genome stability [106]. As an example, in white blood cells, an inverse association between the Alu element methylation level and DNA damage has been observed [107]. Therefore, it has been proposed that Alu methylation plays a crucial role in reducing the accumulation of endogenous DNA damage by minimizing the torsional force on the DNA double helix through naturally occurring gaps in hypermethylated DNA [51]. In accordance with these considerations, Thongsroy et al. observed an inverse association between Alu methylation and fasting glucose levels or HbA1c in patients with T2D compared to healthy controls [51,77]. Recently, we reported no correlation between Alu methylation and glycemic status (HbA1c or fasting glucose) in patients with T1D compared to controls, by using the same method as the previous authors (Alu-COBRA analysis), but in a smaller patient sample. Moreover, in the same study, a positive correlation between an Alu methylation pattern (mCuC and uCmC) and HbA1c in the group of patients with T1D was observed [64].

Results emerging from studies on LINE-1 methylation and DM remain controversial. Specifically, according to five studies as depicted in Table 1, LINE-1 methylation was inversely associated with higher plasma glucose levels, diabetes, and greater risk for metabolic syndrome (MS) [12,78,79,85]. On the other hand, in two studies, a positive correlation was reported between LINE-1 methylation and hyperglycemia or the presence of diabetes [13,80], and in another two studies, no association was detected [82,83].

These observations highlight a potential association between Alu or LINE-1 hypomethylation and the underlying processes of molecular mechanisms, as prolonged hyperglycemia may induce an imbalance in oxidative production and suppression, contributing to impaired insulin signaling [51]. Therefore, LINE-1 and Alu methylation levels might serve as valuable biomarkers for evaluating the clinical outcomes of hyperglycemia. In addition, these retrotransposons might potentially serve in the future as specific novel indicators for more precise screening and monitoring of diabetes progression.

Moreover, to the best of our knowledge, most studies and observations have so far explored the association of methylation of LINE-1 and Alu retrotransposons with the pathogenesis of T2D, although scientific data about their role in T1D are very limited. Further research, particularly prospective studies, is needed to elucidate the connection between LINE-1 and Alu methylation and the onset and progression of DM.

Interpretation of research findings on this subject merits careful consideration, especially due to the heterogeneity in the study designs and populations. It is critical to highlight the variability in study designs, sample sizes, and methods used in the reviewed studies, which may impact the comparability of the findings. Specifically, the number of research studies in this field is rather limited, particularly those focusing on Alu methylation, and the sample size in these studies was commonly small. In addition, the biological samples varied across different studies and different methods were used to assess methylation in each case. In contrast to studies that used LINE-1 as an index of global DNAm, others assessed global DNAm in different repetitive elements, such as Alu repeats, and used other methods rather than bisulfite pyrosequencing. COBRA analysis is regarded as a highly accurate semi-quantitative methylation method, suitable for detecting more than one CpG site and for providing more accurate information about DNA methylation patterns compared to pyrosequencing [51].

LINE-1 and Alu repeats represent distinct measures of dispersed DNAm and might have a variety of functions. Considering that many mechanisms of methylation remain unknown, numerous questions arise on the validity of the measurements [7]. Quantitative evaluation of DNAm at Alu is approximately one-third to one-fourth of methylation observed at LINE-1, which could imply that the epigenetic modifications at LINE-1 and Alu might measure distinctive traits [11].

Interestingly, the impact of methylation varies depending on its location compared to coding genes. Typically, hypermethylation of the promoter CpG island is linked with the silencing of gene transcription, while hypomethylation of CpG islands is commonly connected with enhanced gene expression. On the contrary, DNA methylation in the bodies of genes might be involved in differential promoter usage, transcription elongation, alternative splicing, and increased gene expression [108]. Therefore, the contradictory findings with various indicators of global DNAm may raise a discussion about the functional utility of DNA methylation measurement.

Also, we must take into consideration that modifications of methylation status of LINE-1 and Alu elements are probably reversible and may be affected by environmental factors and through environment–gene interactions. Most of the studies mentioned above are cross-sectional evaluations, which makes it challenging to determine whether certain epigenetic markers are a cause or a consequence of hyperglycemia and the disease process in patients with DM. Therefore, the evaluation of global DNAm offers a simplified view of epigenetic imbalance, as it does not recognize, either quantitatively or qualitatively, the co-existence of hypermethylation and hypomethylation within different genes in the same cell [11]. Thus, further essays are required to analyze the molecular characteristics of these changes and their connection to disease mechanisms in order to develop methods that can differentiate variations in methylation levels from inherent genomic variability of these elements.

Only the application of sophisticated methods for DNAm analysis, which may be standardized across different laboratories, will provide reliable data that may confirm the importance of retrotransposons on DM. More large-scale, observational studies are required to examine the correlation between altered retrotransposons’s methylation and gene expression in DM and reveal their functionality in the etiopathogenesis of the disease.

## 5. Conclusions and Perspectives

With the advancement of sequencing technologies and computational tools, the investigation of the role of retrotransposons in the pathophysiology of multifactorial diseases, such as DM, has become a focal point of interest for researchers. Existing scientific data of studies evaluating the association of retrotransposons with DM are still controversial, and scientific evidence about their role, especially in T1D pathogenesis, is limited. Therefore, future research using long-read sequencing technology may provide more opportunities for better comprehension in the regulation of retrotransposons and their significance during the course of the disease, promising new therapeutic targets for patient-centered interventions.

## Data Availability

Not applicable.
